# Occupational Exposure to Diesel Particulate Matter in Municipal Household Waste Workers

**DOI:** 10.1371/journal.pone.0135229

**Published:** 2015-08-06

**Authors:** Kyong-Hui Lee, Hye-Jung Jung, Dong-Uk Park, Seung-Hun Ryu, Boowook Kim, Kwon-Chul Ha, Seungwon Kim, Gwangyong Yi, Chungsik Yoon

**Affiliations:** 1 Department of Environmental Health^,^ Graduate School of Public Health, Seoul National University, Seoul, Korea; 2 Health Promotion Center, Catholic Kwandong University, International St. Mary’s Hospital, Incheon, Korea; 3 Department of Environmental Health, Korea National Open University, Seoul, Korea; 4 Graduate School of Public Health Korea University, Seoul, Korea; 5 Occupational Lung Disease Institute, Korea Workers Compensation and Welfare Service, Incheon, Korea; 6 Changwon National University, Changwon, Korea; 7 Keimyung University, Daegu, Korea; 8 Occupational Safety & Health Research Institute, KOSHA, Ulsan, Korea; Telethon Institute for Child Health Research, AUSTRALIA

## Abstract

**Objective:**

The purposes of this study were to determine the following: 1) the exposure levels of municipal household waste (MHW) workers to diesel particulate matter (DPM) using elemental carbon (EC), organic carbon (OC), total carbon (TC), black carbon (BC), and fine particulate matter (PM 2.5) as indicators; 2) the correlations among the indicators; 3) the optimal indicator for DPM; and 4) factors that influence personal exposure to DPM.

**Methods:**

A total of 72 workers in five MHW collection companies were assessed over a period of 7 days from June to September 2014. Respirable EC/OC samples were quantified using the thermal optical transmittance method. BC and PM 2.5 were measured using real-time monitors, an aethalometer and a laser photometer. All results were statistically analyzed for occupational and environmental variables to identify the exposure determinants of DPM.

**Results:**

The geometric mean of EC, OC, TC, BC and PM 2.5 concentrations were 4.8, 39.6, 44.8, 9.1 and 62.0 μg/m^3^, respectively. EC concentrations were significantly correlated with the concentrations of OC, TC and BC, but not with those of PM 2.5. The exposures of the MHW collectors to EC, OC, and TC were higher than those of the drivers (p<0.05). Workers of trucks meeting Euro 3 emission standard had higher exposures to EC, OC, TC and PM 2.5 than those working on Euro 4 trucks (p<0.05). Multiple regression analysis revealed that the job task, European engine emission standard, and average driving speed were the most influential factors in determining worker exposure.

**Conclusions:**

We assessed MHW workers’ exposure to DPM using parallel sampling of five possible indicators. Of these five indicators, EC was shown to be the most useful indicator of DPM exposure for MHW workers, and the job task, European emission standard, and average driving speed were the main determinants of EC exposure.

## Introduction

Diesel engines are the primary power sources for heavy-duty trucks, rail-road locomotives, marine vessels and a variety of off-road heavy equipment used in agriculture, construction and mining because they have a longer life, greater power, better fuel economy and require less maintenance compared to gasoline engines [1]. In Korea, the number of diesel fueled vehicles has increased by 44% from 57,220,000 in 2008 to 82,310,000 in 2012. Data indicate that diesel powered vehicles accounted for 36.72% of the total vehicles used in 2012 [2].

Despite the advantages of diesel engines, they generate pollutants that are characterized as diesel engine exhaust (DEE) [1, 3]. DEE is a complex mixture of gaseous and particle-phase emissions. Gaseous components of DEE include carbon dioxide, oxygen, nitrogen, water vapor, carbon monoxide, nitrogen compounds, sulfur compounds, and numerous low-molecular-weight hydrocarbons. The particulate fractions are defined as diesel particulate matter (DPM). DPM is comprised of respirable particles of which 80–95% are fine particles <2.5 μm [1, 4]. DPM consists of a center core of elemental carbon (EC), which has attached organic compounds comprised of carbon and hydrogen molecules as well as small amounts of sulfate, nitrate, and other elements. The organic compounds are defined as organic carbon (OC) and may comprise 19–43% of the DPM, while the EC content may comprise 50–75% of the DPM [1]. The composition and generation of DEE varies depending on the age of the diesel engine, type of engine, fuel characteristics, driving cycle, and whether the exhaust is filtered. [5, 6].

Recently, the International Agency for Research on Cancer (IARC) reclassified DEE as “carcinogenic to humans (Group 1)” based on sufficient evidence that exposure is associated with an increased risk for lung cancer [7, 8]. The decision was based on a US National Cancer Institute (NCI) and National Institute for Occupational Safety and Health (NIOSH) study that showed exposure-response relationships between respirable elemental carbon exposures and lung cancer mortality in underground miners [8, 9]. The IARC also noted a positive association (limited evidence) with an increased risk of bladder cancer (Group 1) [7].

Besides the adverse health effects of DEE, DPM reduces atmospheric visibility and is known as the second leading contributor to global warming after carbon dioxide [10]. Airborne particulate matter reduces the amount of solar radiation affecting the Earth. The black carbon (BC) of DPM also absorbs visible solar radiation in the atmosphere. According to Jacobson, the magnitude of the direct radiative forcing from BC itself exceeds that due to methane, suggesting that BC controls may be more beneficial than methane controls in terms of preventing warming [11].

With the increased use of diesel engines, concern about occupational exposure to DEE is also increasing. Kauppinen et al estimated that approximately 3 million workers were exposed to DEE in 15 countries of the European Union from 1990 through 1993 [12]. The Occupational Safety and Health Research Institute (OSHRI) of Korea reported that the average number of workers exposed to DEE rose from 261,000 in 1993 to 443,000 in 2012 [13].

Numerous studies have evaluated DPM exposure in various occupations, such as railroad repair and locomotive crews, truck and bus drivers, truck/bus garage mechanics, fire fighters, heavy equipment operators, underground miners, and tunnel construction workers. However, few studies have assessed exposure to DEE emissions for municipal household waste (MHW) workers. In Korea, MHW workers are occupationally exposed to DEE because the trash trucks have diesel-fueled engines and workers generally operate at the rear of the trucks where the tailpipes are located.

It has been reported that MHW workers are potentially exposed to musculoskeletal injury, bioaerosols, infectious materials, temperature extremes, diesel exhaust, and particulate matter [14, 15]. Previous studies on waste handlers have mainly focused on accident and occupational disease prevalence rates, exposures to bioaerosols and the association of bioaerosol exposure to health. Park et al. assessed the size characteristics of particulate matter and the effects of the type of waste-handling activity on the levels of PM during waste collection and sorting. However, the PM samples were not based on the personal exposures during a shift, and the measurements were not specific for DPM [16].

Since DPM is a mixture of various components, EC, OC, total carbon (TC, EC+OC), BC and fine particulate matter (PM 2.5) can be used to determine DPM exposures [17–19]. Among these, EC is known as the most useful marker of DPM because it is generated proportionally to DPM, relatively free of interferences (unlike OC), and can be measured at low concentrations [17, 18, 20]. BC (black aerosol, soot, carbonaceous aerosol) is often used interchangeably with EC, but the term was defined by measuring light-absorbing carbon. EC and BC are comparable but slightly different in thermal, optical, and chemical characteristics.

The purposes of this study were to determine the following: 1) the exposure of MHW workers to DPM using EC, OC, TC, BC and PM 2.5 as indicators; 2) the correlations among these indicators; 3) the optimal indicator for DPM; and 4) factors that influence personal exposure to DPM.

## Materials and Methods

### Exposure Group Selection and Task Description

Five Korean MHW collecting companies, three in Goyang and two in Seoul agreed to participate in this study. Goyang is a medium-sized (267.31 km^2^) suburban city near Seoul with a population of 1 million. Seoul is a metropolitan city with 10 million residents.

In Korea, MHW is classified into three types: solid waste, food waste, and recyclable materials such as plastic, paper, cans, clothes and bottles. All of the companies collect all three types of MHW. Workers who collect recyclable waste were excluded from this study because the recyclable waste trucks use LPG (liquefied petroleum gas). Only MHW workers who use diesel-powered trucks were included in this study. Trucks that collected solid waste went either to their respective incineration plants or to interim collection points such as landfills 2–5 times per day, depending on their route and pick-up locations. The food waste trucks went to their recycling plant several times a day.

**Fig 1:**
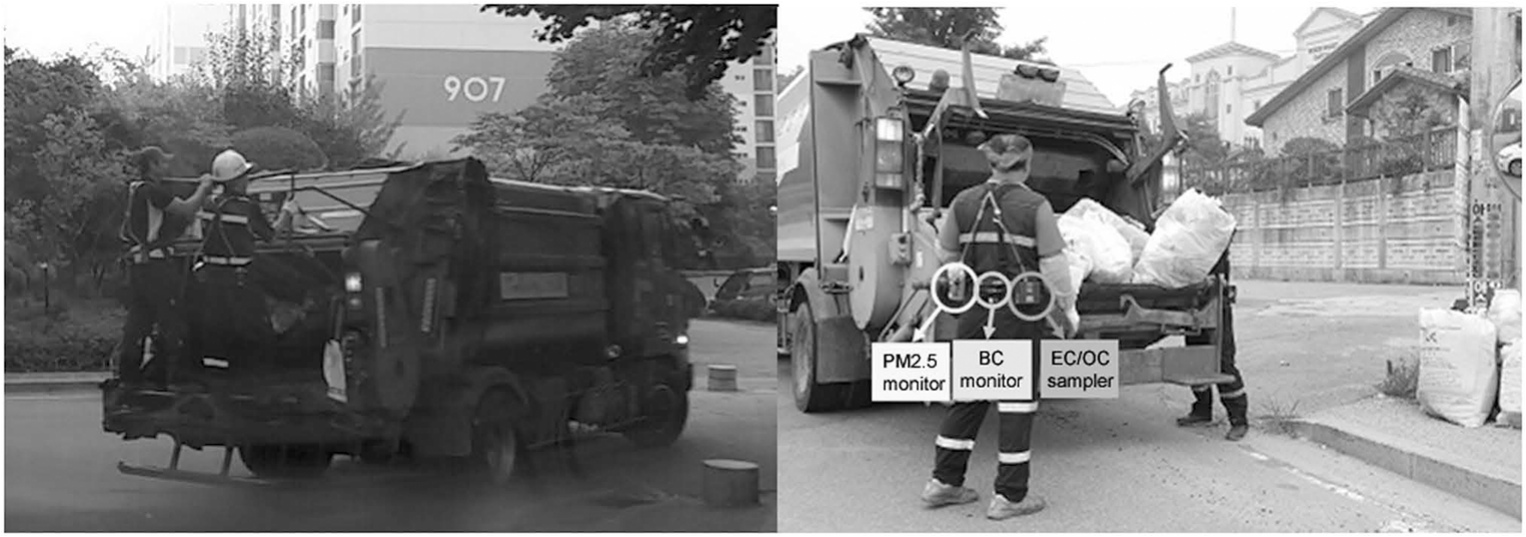
Photographs of municipal household waste-collecting activities. Left: Riding on the rear of a truck. Right: Collecting MHW with samplers mounted.

A MHW collection truck is manned by 1–2 collectors and a driver. Collectors retrieve the MHW and dump it into the rear compartment of the truck. All of the trucks are equipped with a GPS (Global Positioning System) system, and hydrodynamic presses and have semi-automated systems to lift the trash bins or containers to dump the trash into the trucks. Collectors usually stay in the rear of the truck to dump the trash and to operate the press and lifting mechanisms. All of the exhaust tailpipes of the trash trucks are positioned under and toward the rear of the trucks and the rear of the truck is where workers have the greatest risk of exposure to DEE (Fig 1). Drivers stayed inside the trucks for more than 6 hours unless they needed to assist the collectors. Drivers would help if there were only one collector.

### Sampling Strategy

Field sampling was conducted over a period of 7 days between 26 June and 18 September 2014. The sampling locations, dates, number of samples collected and waste type are listed in S1 Table. Seventy-two EC/OC/TC, 17 BC, and 21 PM 2.5 personal samples were collected from 72 MHW workers. Prior to each sampling date, workers and managerial staff were briefed on the plan, purpose, and method of the sampling, and the majority of the workers agreed to participate. Because of the limited number of available instruments for BC and PM 2.5 sampling, just one to two trucks and their workers were selected for comparative sampling of EC/OC/TC, BC and PM 2.5 during the meeting. To minimize possible sampling bias, we selected the most representative ones after discussing the workload, manning, collection route and locations with the company manager and workers.

On the sampling day, all workers who volunteered for sampling wore an EC/OC/TC sampler. The workers who had previously been selected for comparative sampling additionally wore BC and PM 2.5 samplers, as shown in Fig 1. The sampling was performed during the entire workday. Work schedules differed among the companies and between the two cities. The workday also varied depending on the route and the amount of MHW collected. Typically, a workday and sampling period ranged from 400 to 500 minutes. Since MHW collection is physically demanding, we were unable to collect repeat samples from the same worker. After the sampling was completed, all workers answered a short questionnaire about their employment history, number of service years and smoking habits.

### Sampling and Analysis

All samples were collected in the breathing zone of the collectors and drivers. EC/OC/TC samples were collected on 37-mm diameter, pre-fired quartz filters (Pallflex Tissuquartz 2500QAT-UP, Pall Life sciences, USA) mounted on a personal environmental monitor (PEMs, Cat No 761–203, SKC Inc., USA) using a personal sampling pump (MSA Escort ELF pump, Mine Safety Appliance Co., USA). Pumps were pre- and post-calibrated using a DryCal DC-Lite primary flow meter (DCL-H, Bios International Co., USA). According to the PEM manufacturer’s instructions, the pump flow rate was set at 2 L/min. At this rate, PEM samplers have a 50% cut-off point for particulates with an aerodynamic diameter of 2.5 μm. Field blanks were collected daily at the measurement sites and were handled identically to the personal samples. All samples were sent for analysis to the laboratory of the Occupational Lung Diseases Institute, Korea Worker’s Compensation and Welfare Service. This is the only laboratory in Korea that analyzes EC/OC/TC samples using NIOSH method 5040. The laboratory participates in the American Industrial Hygiene Association (AIHA) Proficiency Analytical Testing (PAT) program. 1.5 cm^2^ of the quartz filter was punched out and analyzed using an OCEC carbon aerosol analyzer (Sunset Laboratory Inc., USA). The limit of detection (LOD) was 0.2 μg per cm^2^ filter for both EC and OC. All sample measurements for this study exceeded the detection limit.

BC was measured using an aethalometer (microAeth model AE51, Magee Scientific, USA). This instrument measures the intensity of light (880 nm wavelength) transmitted through a T60 Teflon coated glass fiber and reports BC concentrations in ng/m^3^. The default manufacture’s specific attenuation coefficient of 16.6 m^2^/g was used. The air sampling rate was set at 0.15 L/min to enhance the sensitivity per the manufacturer’s manual. Real-time measurements were recorded every minute.

The PM 2.5 concentrations were measured using a real-time laser photometer (SidePak Model AM510, TSI Inc., USA). The SidePak has a built-in PM 2.5 μm impactor. The instrument was set to an airflow rate of 1.7 L/min. All SidePaks used had been calibrated by the manufacturer within the recommended one year interval. Real-time readings were collected every minute. The measured PM levels were corrected using the gravimetric calibration factor, which was determined by collecting parallel samples on PVC filters (37-mm, pore size 5.0 μm, SKC, Inc., USA) mounted on the PEM samplers. Detailed experimental procedures for the determination of the calibration factor are presented in the S1 File.

### Ambient Background Levels

Ambient concentrations of EC, OC, TC, BC and PM 2.5 were obtained from the air pollution monitoring stations in Goyang and Seoul and were taken from the database of Air Quality Information of Seoul metropolitan area and GyeongGi-Do. Monitoring stations are located on the roofs of 3‒4 story buildings in residential areas and near main streets. The monitoring station data used in our study were located where the MHW workers made their collections. However, if there was no monitoring station near the collection site, then the data from the closest station were used for the background values.

The Air Quality Information monitors use a semi-continuous OCEC field instrument (Sunset Laboratory Inc., USA) for EC/OC/TC, and aethalometer (model AE22, Magee Scientific Company, USA) for BC. PM 2.5 concentrations were measured by a ß-ray absorption method using a continuous particulate analyzer (SPM 613-D, Kimoto, Japan). All measurements were collected at hourly intervals and the mean concentrations were calculated from the sampling period.

### Statistical Analysis

Probability plots of EC/OC/TC, BC and PM 2.5 data were right-skewed and a Kolmogorov-Smirnov analysis of the data indicated that the measurements would be best described by a lognormal distribution. All time-weighted average (TWA) data were natural-log-transformed for statistical analysis, and the geometric mean and geometric standard deviation were used for the mean and standard deviation in the descriptive statistics. Although real-time measurements were made for the PM 2.5 and BC monitors, only TWA values were used in this study. The real-time measurements will be described in later article. The descriptive statistics (geometric mean, geometric standard deviation, minimum and maximum) were calculated. A Pearson’s correlation analysis was performed to assess the relationships among the log-transformed concentrations of each DPM indicator.

All EC/OC/TC, BC and PM 2.5 results were classified using environmental and occupational variables such as job task (collector vs. driver), waste type (solid vs. food), age of the diesel vehicle (1‒5 yrs, 6‒10 yrs and 11‒15 yrs), diesel engine emission standard (Euro 3 vs. Euro 4), truck payload capacity (≤2.5 ton vs. 5 ton), diesel particulate filter (DPF) (factory-installed vs. retrofitted), location (suburban vs. urban), number of collected truck containers (1‒2 vs. 3‒4) and worker smoking habits (smoker vs. non-smoker). An analysis of variance (ANOVA) and t-test were used to evaluate the variability within and between the categories of occupational and environmental variables and to compare average levels among categories of occupational and environmental variables.

Multiple regression analysis was performed to identify the main exposure determinants for EC and OC. Categorical variables with p-value <0.05 in the ANOVA were included in a multiple regression analysis. In addition, continuous variables were investigated using univariate analysis, and significant variables with p-value<0.05 entered into a multiple regression analysis. The categorical variables analyzed were job task (collector vs. driver), waste type (solid vs. food), diesel engine emission standard (Euro 3 vs. Euro 4), DPF (factory-installed vs. retrofitted), smoking habits (smoker vs. non-smoker), city (Goyang vs. Seoul), and location (suburban vs. urban). The continuous variables analyzed were driving distance (km), average driving speed (km/h), and percentage of slow driving (< 20 km/h) during the sampling period, weight of collected waste (tons), truck age (y), and truck payload capacity (tons). A multiple linear regression model with the backward elimination method was used. For the final models, differences were considered significant at p<0.05. Model diagnostics were performed with plots of residuals against predicted values and using standardized normal probability plots. Statistics analysis was performed using SPSS 20.0 software (IBM, Armonk, NY).

## Ethics Statement

This work was approved by the Institutional Review Board (IRB) of Chang Won University (IRB approval no: 1040271-201409-HR-012). Written and oral information was presented to all participants by the research staff. The individual in this manuscript has given written informed consent (as outlined in PLOS consent form) to publish these case details.

## Results

A total of 72 EC/OC/TC, 17 BC and 21 PM 2.5 measurements were made during MHW collections of solid and food waste. Table 1 shows the TWA values for EC, OC, TC, BC and PM 2.5 for each company. The TWA values for each worker are presented in S2 File. None of the EC and OC measurements were below substance analytical LODs. All measurements were higher than the ambient background levels. The average ratio of exposure level to background level for EC, OC, TC, BC and PM 2.5 was 4.1, 12.7, 9.8, 2.0 and 4.4, respectively. Ambient background levels are shown in Table 1 and the background levels for each day of sampling are listed in S2 Table.

**Table 1:** Exposure levels (μ/m^3^) of EC, OC, TC, BC and PM 2.5 by company.

City	Company	Sampling date	EC		OC^1)^	TC^1)^	BC		PM2.5^1)^	
			N	GM (GSD)	GM (GSD)	GM (GSD)	N	GM (GSD)	N	GM (GSD)
				(Range)	(Range)	(Range)		(Range)		(Range)
Goyang	A	6/26/2014	12	5.8 (2.2)	56.1 (1.3)	63.1 (1.4)	3	9.7 (1.1)	3	125.0 (1.4)
				(2.3–29.0)	(33.3–97.8)	(35.7–115.0)		(8.4–11.0)		(98–188)
	B	7/1, 2	34	4.8 (1.7)	44.5 (1.5)	48.9 (1.5)	6	9.4 (1.5)	5	102.7 (1.9)
		&11/2014		(2.4–22.3)	(20.6–107.8)	(23.3–112.2)		(6.3–19.6)		(54–240)
	C	7/10/2014	12	4.1 (1.3)	29.2 (1.4)	33.4 (1.4)	2	7.2 (1.2)	4	49.9 (1.5)
				(2.4–6.4)	(19.4–52.3)	(22.9–58.5)		(6.5–8.0)		(33–78)
	Subtotal		58	4.8 (1.7)	42.8 (1.5)	47.6 (1.5)	11	9.0 (1.4)	11	84.8 (1.8)
				(2.3–29.0)	(19.4–107.8)	(22.9–115.0)		(6.3–19.6)		(33–240)
	Background Level^2)^			1.6	3.9	5.5		5.9		23.0
Seoul	D	9/16/2014	8	3.4 (1.5)	34.9 (1.5)	38.7 (1.5)	4	7.5 (1.2)	5	36.8 (1.1)
				(1.7–5.2)	(22.3–69.5)	(24.0–72.2)		(6.1–9.2)		(33–44)
	E	9/18/2014	6	7.1 (1.7)	22.5 (1.6)	30.1 (1.6)	2	13.9 (1.0)	4	46.7 (1.6)
				(3.5–14.2)	(13.5–53.7)	(17.0–63.2)		(13.6–14.2)		(27–71)
	Subtotal		14	4.7 (1.8)	28.9 (1.6)	34.8 (1.5)	6	9.2 (1.4)	9	40.9 (1.4)
				(1.7–14.2)	(13.5–69.5)	(17.0–72.2)		(6.1–14.2)		(27–71)
	Background Level			2.2	4.5	6.7		3.7		13.6
Total Samples			72	4.8 (1.7)	39.6 (1.6)	44.8 (1.5)	17	9.1 (1.4)	21	62.0 (1.9)
				(1.7–29.0)	(13.5–107.8)	(17.0–115.0)		(6.0–19.6)		(27–240)

^1)^ p<0.05, OC, TC and PM 2.5 levels were significantly different by city.

^2)^ Ambient background level for each sampling day is presented in the S2 Table.

Abbreviations: EC: elemental carbon; OC: organic carbon; TC: total carbon; BC: black carbon; PM 2.5: particulate matter 2.5

Filter samples of EC TWAs ranged from 1.7 to 29.0 μg/m^3^ with a geometric mean of 4.8 μg/m^3^ and the OC TWAs ranged from 13.5 to 107.8 μg/m^3^ with a mean of 39.6 μg/m^3^. Real-time measurements for BC had TWAs that ranged from 6.0 to 19.6 μg/m^3^ with a mean of 9.1 μg/m^3^. The real-time measurement TWAs for PM 2.5 ranged from 27 to 240 μg/m^3^ with a mean of 62 μg/m^3^. T-test results showed that the OC and PM 2.5 levels were significantly different between Goyang and Seoul (p<0.05), but the EC and BC levels were not significantly different.

### Relationships between DPM concentrations and various exposure factors

Table 2 presents a comparison of the EC, OC, TC, BC and PM 2.5 concentrations among occupational and environmental categories. The mean EC (N = 42, 5.6 μg/m^3^), OC (44.2 μg/m^3^), and TC (50.1 μg/m^3^) for MHW collectors were significantly higher than those for drivers (EC, N = 30, 3.8 μg/m^3^, p = 0.003; OC, 34.1 μg/m^3^, p = 0.015; TC, 38.3 μg/m^3^, p = 0.008). This indicates that the job task significantly influenced personal exposure levels of EC, OC and TC. Similarly, the mean BC (N = 10, 10.1 μg/m^3^) and PM 2.5 (N = 11, 68.6 μg/m^3^) for the collectors were slightly higher than those of the drivers (BC, N = 7, 7.8 μg/m^3^ and PM 2.5, N = 10, 55.6 μg/m^3^), albeit not significantly so.

**Table 2:** EC, OC, TC, BC and PM 2.5 levels (μ/m^3^) according to occupational and working environment factors.

Factor		EC			OC		TC		BC			PM 2.5		
		N	GM (GSD)	p-value	GM (GSD)	p-value	GM (GSD)	p-value	N	GM (GSD)	p-value	N	GM (GSD)	p-value
Job task	Collector	42	5.6 (1.8)	**0.003**	44.2 (1.6)	**0.015**	50.1 (1.5)	**0.008**	10	10.1 (1.4)	0.108	11	68.6 (2.0)	0.456
	Driver	30	3.8 (1.5)		34.1 (1.5)		38.3 (1.5)		7	7.8 (1.3)		10	55.6 (1.8)	
Waste type	Solid	55	5.0 (1.8)	0.281	39.5 (1.5)	0.934	45.0 (1.5)	0.881	17	9.1 (1.4)	-	20	62.1 (1.9)	1.000
	Food	17	4.2 (1.5)		40.0 (1.7)		44.2 (1.7)		-	-		1	62.0 (—)	
Truck age	1–5yrs	22	4.0 (1.5)	0.178	36.4 (1.7)	0.269	41.1 (1.6)	0.297	9	9.3 (1.3)	0.780	11	52.9 (1.9)	0.398
	6–10yrs	21	5.0 (1.9)		37.5 (1.5)		42.9 (1.5)		4	9.4 (1.6)		6	66.7 (2.0)	
	11–15yrs	29	5.3 (1.8)		44.0 (1.5)		49.3 (1.5)		4	8.2 (1.3)		4	86.4 (1.6)	
Engine emission standard	Euro 3	41	5.6 (1.9)	**0.004**	45.0 (1.5)	**0.005**	50.8 (1.5)	**0.004**	6	9.4 (1.5)	0.764	6	96.2 (1.8)	**0.037**
	Euro 4	31	3.9 (1.5)		33.5 (1.6)		38.0 (1.5)		11	8.9 (1.3)		15	52.1 (1.7)	
Truck payload capacity	≤2.5ton	16	4.7 (1.7)	0.908	29.9 (1.6)	**0.004**	35.7 (1.5)	**0.016**	6	9.2 (1.4)	0.911	9	40.9 (1.4)	**0.004**
	5ton	56	4.8 (1.8)		43.0 (1.5)		47.8 (1.5)		11	9.0 (1.4)		12	84.8 (1.8)	
Diesel Particulate Filter ^2)^	Factory installed	40	4.3 (1.7)	0.213	36.0 (1.6)	0.107	40.9 (1.5)	0.117	13	9.4 (1.4)	0.473	17	57.4 (1.9)	0.246
	Retrofitted	30	5.5 (1.4)		45.3 (1.5)		50.8 (1.5)		4	8.2 (1.3)		4	86.4 (1.6)	
Distance to tailpipe ^1)^	<4m	33	6.3 (1.8)	**0.010**	44.2 (1.6)	0.996	50.7 (1.6)	0.759	7	10.3 (1.4)	0.721	7	66.8 (2.2)	0.875
	≥4m	9	3.6 (1.3)		44.2 (1.5)		48.2 (1.4)		3	9.5 (1.2)		4	71.9 (1.7)	
Location	Suburban Area	18	4.2 (1.4)	0.266	36.2 (1.5)	0.332	40.1 (1.5)	0.215	5	8.9 (1.2)	0.852	5	91.9 (1.5)	0.109
	Urban Area	54	5.0 (1.8)		40.9 (1.6)		46.4 (1.6)		12	9.2 (1.4)		16	54.9 (1.9)	
# of collected truck containers	1–2	40	4.1 (1.7)	**0.007**	40.2 (1.7)	0.790	44.6 (1.7)	0.935	9	8.4 (1.2)	0.309	10	79.3 (1.8)	0.086
	3–4	32	5.8 (1.7)		39.0 (1.4)		45.0 (1.4)		8	9.9 (1.5)		11	49.7 (1.8)	
Smoking ^2)^	Smoker	40	4.9 (1.8)	0.866	49.3 (1.4)	**<0.001**	54.5 (1.4)	**<0.001**	7	8.9(1.5)	0.877	12	68.7 (2.0)	0.656
	Non smoker	30	4.7 (1.7)		30.6 (1.5)		35.4 (1.5)		10	9.2 (1.3)		9	60.5 (1.8)	
Total		72	4.8 (1.7)		39.6 (1.6)		44.8 (1.5)		17	9.1 (1.4)		21	62.0 (1.9)	

^1)^ Straight distance from the end of tailpipe to the back end of the truck, where MHW collectors mainly stay. Drivers are not included in this category.

^2)^ There are two missing values.

Abbreviations: EC: elemental carbon; OC: organic carbon; TC: total carbon; BC: black carbon; PM 2.5: particulate matter 2.5

All MHW trucks surveyed had manufacture dates after 2000. Their average age was 8.2 y and they met either the Euro 3 or 4 diesel engine emission control standards. The ANOVA analysis results indicated that the engine emission standard (Euro 3 vs. Euro 4) was a significant factor affecting personal exposure levels to EC, OC, TC and PM 2.5, whereas the age group of the truck was not. The workers using Euro 3 Standard trucks were exposed to significantly higher levels of EC (N = 41, 5.6 μg/m^3^, p = 0.004), OC (45.0 μg/m^3^, p = 0.005), TC (50.8 μg/m^3^, p = 0.004) and PM 2.5 (N = 6, 96.2 μg/m^3^, p = 0.037) than those working on the Euro 4 trucks (EC, N = 31, 3.9 μg/m^3^; OC, 33.5 μg/m^3^; TC, 38.0 μg/m^3^; PM 2.5, N = 15, 52.1 μg/m^3^). Those working on trucks with a payload capacity equal to 5 tons had significantly higher exposures to OC (29.9 vs. 43.0 μg/m^3^, p = 0.004), TC (35.7 vs. 47.8 μg/m^3^, p = 0.016) and PM 2.5 (40.9 vs. 84.8 μg/m^3^, p = 0.004) than those on trucks with a payload capacity of less than 2.5 tons. No such relationship was found for the EC and BC data.

All of the exhaust tailpipes of the MHW trucks were positioned under and toward the rear of the trucks. The distance from the tailpipe to the rear of the truck varied from 1.2 to 4.2 m, depending on the truck model. The newer trucks had greater distances between the tailpipe and the rear of the truck. The collectors working on trucks with greater distances (≥4 m) between the tailpipe and the rear of the truck had lower EC exposures than the collectors who worked on trucks that had tailpipes closer than 4m to the rear of the truck (6.3 vs. 3.6 μg/m^3^, p = 0.010). However, this relationship was not observed for OC, TC, BC and PM 2.5 measurements.

The number and quantity of waste of the collections was a significant factor for the DPM exposure levels. Workers who collected more containers (3‒4 vs. 1‒2) had significantly higher exposure levels to EC (5.8 vs. 4.1 μg/m^3^, p = 0.007), but there was no significant difference for OC, TC, BC and PM 2.5. The workers who smoked during the sampling period had mean exposures to OC (49.3 vs. 30.6 μg/m^3^, p<0.001) and TC (54.5 vs. 35.4 μg/m^3^, p<0.001) that were significantly higher than those of the non-smokers, but there was no significant difference in their exposures to EC, BC and PM 2.5.

**Fig 2:**
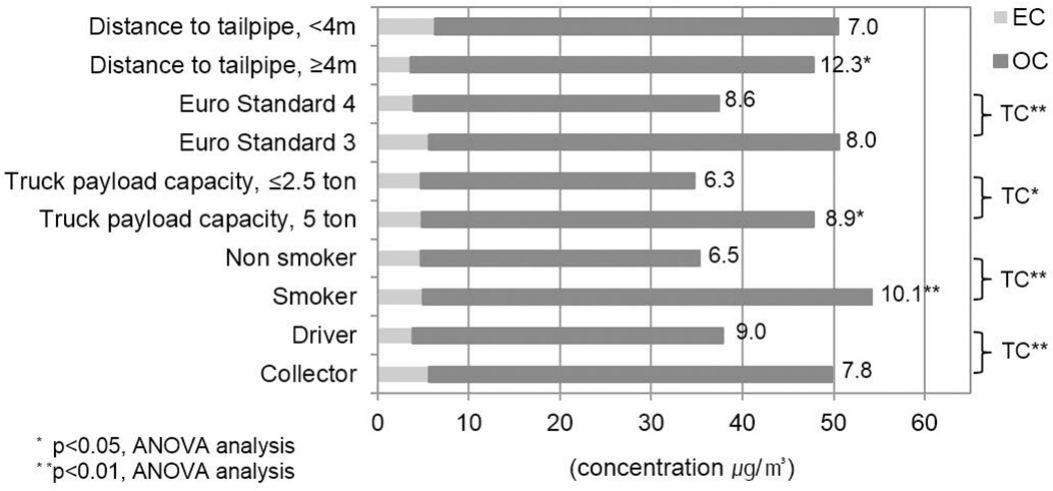
Geometric mean of TC and OC/EC according to job task, smoking habit, and type of truck. The geometric mean TC is presented as a bar chart. Each bar is the sum of EC (ivory bar) and OC (gray bar). The mean levels of TC for the collectors, smokers, workers on larger trucks and on trucks meeting Euro Standard 3 were significantly higher than those of the drivers, non-smokers, workers on smaller trucks, and those working on trucks meeting Euro Standard 4. The OC/EC is shown at the end of each bar. The OC/EC ratios for smokers, workers on larger trucks, and workers on trucks that had greater distances between the tailpipe and rear of the truck were significantly higher than those for the other categories of workers.

Fig 2 shows plots of mean TC levels for job tasks, smoking habits and vehicle factors. The mean levels of TC for the collectors, smokers, workers on larger trucks, and on trucks meeting Euro Standard 3 were significantly higher than the levels of the drivers, non-smokers, workers on smaller trucks, and those working on trucks meeting Euro Standard 4. Fig 2 also shows the ratio of OC to EC at the end of each column, which ranged from 1.4 to 26.1, with a mean of 8.2. The mean ratio of OC to EC for smokers, workers on larger trucks, and workers on trucks that had greater distances between the tailpipe and rear of the truck was significantly higher than those for the other categories of workers. This indicates that the former workers were exposed to significantly higher fractions of OC compared to EC.

### Correlations between DPM indicators

The concentrations of EC were significantly correlated with the concentrations of OC, TC and BC, indicating a consistent pattern among representative DPM indicators (Table 3). The Pearson correlation coefficients between EC levels and OC, TC, and BC were 0.325 (p<0.01), 0.468 (p<0.001), and 0.822 (p<0.001), respectively. PM 2.5 levels showed significant correlations with OC and TC, but not with EC and BC. Since TC is the sum of EC and OC, the significant correlation between PM 2.5 and TC is also related to the correlation between OC and PM 2.5.

**Table 3:** Correlation coefficients among levels of EC, OC TC, BC and PM 2.5.

	EC	OC	TC	BC	PM2.5
EC	1.000				
OC	0.325^1)^	1.000			
TC	0.468^3)^	0.983^3)^	1.000		
BC	0.822^3)^	0.258	0.458^2)^	1.000	
PM2.5	0.283	0.650^1)^	0.677^3)^	0.319	1.000

^1)^ p<0.01 correlation is significant at the 0.01 level (one-tailed).

^2)^ p<0.05, correlation is significant at the 0.05 level (one-tailed).

^3)^ p<0.001 correlation is significant at the 0.001 level (one-tailed).

Abbreviations: EC: elemental carbon; OC: organic carbon; TC: total carbon; BC: black carbon; PM 2.5: particulate matter 2.5

### Multiple linear regression analysis

Table 4 summarizes the results of the multiple linear regression analysis performed to identify exposure determinants affecting the levels of EC and OC. The EC multiple regression model included seven variables related to the vehicle, worker activity, and environment. The factors included in the multiple regression analysis were selected after performing a univariate analysis using a significance level of 0.05. The univariate analysis results were: job task (ß = 0.387, p = 0.003), Euro engine emission standard (ß = -0.376, p = 0.004), truck age (ß = 0.043, p = 0.024), number of truck containers collected (ß = 0.267, p = 0.008), percentage of slow driving (< 20 km/h) during the sampling period (ß = 2.146, p = 0.014), average driving speed (ß = -0.038, p = 0.024), and the ambient background level (ß = 0.043, p = 0.682). The background level was applied to adjust for the effects of ambient levels. Ambient values vary depending on the amount of traffic and the occasional Asian dust event [21, 22]. The variables were selected based on the backward elimination method for the multiple regression model. The final model to predict EC exposure levels included job task, Euro engine standard, and average driving speed (adjusted R^2^ = 0.382, p<0.001).

**Table 4:** Multiple regression models to predict natural log-transformed EC and OC levels.

		EC level, μg/m^3^			OC level, μg/m^3^		
Independent factors		Coefficient	Standard error	p-value	Coefficient	Standard error	p-value
Job title	Driver	Reference	0.122	0.001	Reference	0.081	0.014
	Collector	0.408			0.205		
Diesel exhaust emissions standard	Euro Standard 3	Reference	0.127	<0.001	Reference	0.084	0.003
	Euro Standard 4	-0.536			-0.256		
Average driving speed		-0.055	0.014	<0.001			
Truck payload capacity					0.089	0.038	0.025
Smoking	Nonsmoker				Reference	0.08	<0.001
	Smoker				0.46		
Ambient background level		0.109	0.096	0.262	-0.039	0.037	0.288
Intercept		2.576	0.37	<0.001	3.169	0.267	<0.001
Modeling Results	Adjusted R^2^	0.382	0.439	<0.001	0.47	0.328	<0.001

Six variables were included in the OC model; job task (ß = 0.261, p = 0.015), Euro engine emission standard (ß = -0.295, p = 0.005), truck payload capacity (ß = 0.140, p = 0.004), smoking (ß = 0.094, p<0.001), city (ß = -0.397, p = 0.003), and ambient background level (ß = -0.063, p = 0.198). The final model to predict the OC exposure level included smoking, Euro engine standard, job task and truck payload capacity (adjusted R^2^ = 0.470, p<0.001).

## Discussion

Our study determined the exposure levels of MHW workers to DPM by sampling and analyzing EC, OC, TC, BC and PM 2.5. All measurements were considerably higher than the ambient background levels; the mean ratio of exposure levels to background levels for EC, OC, TC, BC and PM 2.5 were 4.1, 12.7, 9.8, 2.0 and 4.4, respectively. Among the five indicators, EC measurements showed consistent and reliable exposure patterns against various exposure factors, such as job task, European engine emission standard, distance from the rear of the truck to the engine tailpipe, age of truck, average driving speed, number of containers collected, etc. The concentrations of EC were significantly correlated with the concentrations of OC, TC and BC indicating a consistent pattern among representative DPM indicators. The multiple regression model confirmed that job task, European engine emission standard and average driving speed were the most influential factors in determining EC exposures.

We assessed personal exposure levels to EC, OC, TC, BC and PM 2.5 for five MHW collection companies. However, there is no occupational exposure limit (OEL) for DPM recommended by standard occupational safety organizations for general industry workers. The existing OEL guidelines are all for underground miners. The Mine Safety and Health Administration (MSHA) recommends a Permissible Exposure Limit (PEL) of 160 μg/m^3^ (measured as TC which is equivalent to 120 μg/m^3^ for EC) while the Australia Department of Natural Resources and Mines (DNRM) recommends an exposure limit of 100 μg/m^3^ (measured as EC)[23, 24]. The MSHA states that its PEL was based on feasible control of emissions in mines and not on adverse health effects. American Conference of Governmental Industrial Hygienists (ACGIH) proposed a Notice of Intended Change (NIC) of the Threshold Limit Value (TLV) to 20 μg/m^3^ expressed as EC in 2001, but withdrew the NIC in 2003. Some local governments use a guideline of 20 μg/m^3^ [25]. This is the lowest OEL value recommended for general industry workers [26]. In this study, three of 72 EC measurements (4.1%) exceeded the 20 μg/m^3^ concentration and the 95^th^ percentile of EC measurements was 22.0 μg/m^3^ for MHW collectors and 10.0 μg/m^3^ for drivers.

Comparisons of DPM levels should be carefully examined because of differences in sampling and analytical methods for the DPM indicators. Several sampling and analysis methods have been used to measure EC concentrations and the results can significantly differ depending on the measurement technique [27–30]. Nevertheless, it is clear that the exposure levels of MHW workers were markedly lower than those for underground miners and tunnel construction workers [31, 32]. Table 5 presents the exposure levels to EC of other occupational groups measured using NIOSH method 5040. Compared with other studies, the MHW collectors (GM = 5.6 μg/m^3^) were exposed to slightly higher levels than mechanics of truck repair garages (GM = 3.2–5.9 μg/m^3^), mechanics of locomotive workshops (GM = 2.6–3.2 μg/m^3^), truck drivers (GM = 1.1–4.0 μg/m^3^), railroad crews (GM = 1.4–5.6 μg/m^3^), and surface workers at mining facilities (GM = 1–4 μg/m^3^) [18, 20, 31, 33–37]. The exposures of MHW truck drivers (GM = 3.8 μg/m^3^) were comparable to local truck drivers (GM = 1.2–4.0 μg/m^3^) and long-haul truck drivers (GM = 1.1–3.8 μg/m^3^).

**Table 5:** Occupational exposure levels to EC for different occupational groups.

**Occupational groups**		**Agent**	**N**	**GM**	**GSD**	**Location**	**Reference**
Drivers	Truck drivers, local	EC_S_	576	1.2	2.8	US	Davis et al., 2007
	Truck drivers, local	EC_I_	-	4	-	US	Liukonen et al., 2002
	Truck drivers, long haul	EC_S_	349	1.1	2.3	US	Davis et al., 2007
	Truck drivers, long haul	EC_I_	-	3.8	-	US	Liukonen et al., 2002
	Bus drivers	EC_S_	39	1.4	3.3	US	Ramachandran et al., 2005
Ramp attendants		EC_S_	34	1.1	1.8	US	Ramachandran et al., 2005
Mechanics	Garage mechanics	EC_S_	35	3.2	1.7	US	Ramachandran et al., 2005
	Truck mechanics	EC_I_	40	5.9	3.1	Canada	Seshagiri et al., 2003
	Locomotive workshop worker	EC_I_	40	2.6	3.2	Canada	Seshagiri et al., 2003
	Railway Mechanics	EC_R_	28	3.2	2.4	Canada	Verma et al., 2003
Railroad crews	Lead locomotives	EC	156	1.4	3.2	US	Hewett et al., 2014
	Trailing locomotives	EC	22	5.6	3.4	US	Hewett et al., 2014
	Train driver	EC_R_	23	2.3	2	Canada	Verma et al., 2003
	Lead locomotives (without preceding stacks)	EC_I_	33	2.5	1.5	US	Liukonen et al., 2002
Fire fighters		EC_Ia_	16	1.5	-	US	Roegner et al., 2002
		EC_I_	12	<LOQ, 16(max)	-	US	NIOSH, 1998
Mining surface workers	Limestone facility	EC_R_	33	4	2.2	US	Coble et al., 2010
	Potash facility	EC_R_	61	1	3.9	US	Coble et al., 2010

Abbreviations: EC_S_: submicron elemental carbon; EC_R_: respirable elemental carbon; EC_I_: inhalable elemental carbon; EC_Ia_: area sample of EC_I_; LOQ: limit of quantification

Ambient background levels could be an important factor when comparing the exposure levels of MHW workers to those of other occupations. MHW workers start their work either early in the morning or at night to avoid traffic congestion. Some of the sampling periods included travel during the morning rush hour. No Asian dust or yellow sand events, which can significantly affect the sampling results, occurred during the sampling dates. Our results indicated that MHW workers had occupational exposures to DPM that were much higher than ambient background levels (p<0.001).

BC levels showed high correlation with EC levels, but the mean BC level was about twofold the mean EC level. The ratio of BC/EC ranged from 1.18 to 3.08 with a mean of 1.99. Numerous inter-method and inter-location comparisons to determine EC and BC levels showed variations between EC and BC concentrations [38–42]. Since BC measurements contain organic components that absorb light (e.g., brown carbon) in addition to EC, this may explain the differences between EC and BC [38, 41]. An inter-comparison study of BC and EC levels reported that the slopes of co-located BC vs. EC measurements were 2.7 and 3.3 for two cities during the summer of 2002 [40]. A study performed in Korea on seasonal variations in BC and EC levels in the atmosphere, reported ratios of BC/EC ranging from 0.98 to 1.38, showing the highest slope in the summer of 2007 [39]. It has been reported that the optical attenuation coefficients to measure BC can differ depending on the size distribution and mixing state of the aerosols, chemical characteristics of light-absorbing species, and deposited mass per unit time [40, 43–45]. Since the aethalometer uses a specific attenuation coefficient of 16.6 m^2^/g, BC measurements using aethalometer for EC require site-specific calibration because of the optical properties of the aerosol.

One-way ANOVA and univariate analysis showed that EC levels were significantly related with the truck engine’s European emission standard, job task, average truck speed, distance from the rear of truck to tailpipe, age of truck, and workload (number of containers collected). Among these variables, the multiple regression model confirmed that the engine emission standard, job task, and average truck speed played a key role in the measured EC levels. Although BC levels had the highest correlation with EC, BC levels did not show as significant a relationship or exposure pattern as the EC values. The aethalometer has been rarely used in the industrial hygiene field to evaluate DPM concentrations, but has been used frequently to monitor atmospheric DPM levels. This study indicates that further studies are required to evaluate the validity of BC measurements as a DPM indicator for the occupational environment.

The average PM 2.5 level was 62 μg/m^3^, ranging from 27 to 240 μg/m^3^. These levels were 4.4-fold higher than the ambient background level and higher than the Korean Ministry of the Environment ambient air quality standard for PM 2.5 (24-h average = 50 μg/m^3^, annual average = 25 μg/m^3^) [46]. Park et al. categorized MHW collection activities in Korea and reported PM 2.5 exposure levels during collection (73.29 μg/m^3^), transfer (223.39 μg/m^3^), sorting (61.57 μg/m^3^) and transport (73.90) [16]. Since “transfer” and “sorting” activities are part of recyclable material collection, they were not included in the present study. Our PM 2.5 results were similar to those of the “collection” and “transport” activities in the study by Park et al.

Those workers using Euro 3 Standard engines (p = 0.037) and larger trucks (p = 0.004) were exposed to higher PM 2.5 concentrations. Greater workload (p = 0.086) and operating in suburban areas (p = 0.109) also contributed to higher PM 2.5 concentrations without significance. However, EC levels did not differ based on truck size (p = 0.908) and location (p = 0.266). These results indicate that particulate matter may have originated from other sources besides MHW truck exhaust. Goyang is a medium-sized suburban city that has some unpaved roads, a lake, and an incineration plant within 6 km of the truck routes. These factors may have increased the PM 2.5 concentration in Goyang.

The exposures of the MHW workers had significantly higher OC/EC ratios than those reported in other studies, ranging from 1.4 to 26.1, with a mean of 8.2. It has been reported that the OC/EC ratio is generally <1 for diesel engines and >1 for gasoline engines [47]. In addition, it has been reported that OC interferences should be suspected if the EC/TC ratio is <0.35 [48, 49]. Converting the EC/TC ratio of 0.35 to an OC/EC ratio, if the OC/EC ratio is >1.8, this indicates an additional possible source of OC. We employed a PEM sampler to exclude EC interference by larger particles. However, OC interferences caused by bioaerosols from food and solid waste and cigarette smoke could not be excluded on the basis of their particle size. The multiple regression model for OC confirmed that OC levels were most affected by the workers’ smoking habits.

There is another possible explanation as to why our results showed much higher OC/EC ratios. It has been reported that the chemical composition of the filter and collected sample can influence the temperature at which EC is evolved during thermal-optical analysis [50]. For example, biomass smoke contains inorganic components that catalyze oxidation of EC and result in lowering the oxidation temperature [51]. Wang et al. studied the effect of metal salts on the quantification of EC and OC in DPM and showed that metals in ambient aerosols reduced the oxidation temperature of EC and enhanced the charring of OC, and that the resulting EC/OC ratio was reduced by ~80%; i.e., the OC/EC ratio was increased by fourfold, depending on the metals and metal to carbon ratio [52]. Since MHW workers can be exposed to various trace metals that can be generated from dirt and solid and food wastes, this could explain the high OC/EC ratios and the possibility of underestimation of EC.

Trash trucks manufactured after 2005 had an original factory-installed DPF and trucks manufactured before 2005 were retrofitted at commercial workshops, as required by Korean environmental regulations [53]. There was no significant difference in EC, OC, TC, BC and PM 2.5 levels between factory installed and retrofitted filter systems. DPF is an important factor in the generation of DPM and worker exposure. According to the DPF manufacturer’s specifications, the DPF installed on trucks can reduce DPM by 90% via catalyst reaction and exhaust filtering. This figure is based on the truck being driven for more than 20 minutes at speeds of at least 70 km/h or more. However, the average driving speed for all the MHW trucks surveyed ranged from 11 to 30.2 km/h where the driving period over 70 km/h was less than 6 min/day. Therefore, the efficiency of the DPFs would be expected to be considerably lower than the manufacturer’s claim.

Several limitations are associated with this study, and it might not be sufficiently representative of MHW collection. MHW collection varies according to the size and location of the routes, the trucks, emissions controls, local environmental conditions, waste management system, etc. The number of parallel samples collected was small due to the limited number of instruments for BC and PM 2.5, and fewer BC and PM 2.5 samples were collected than EC and OC samples. The BC and PM 2.5 data showed some concentration differences depending on the job task and number of collected containers, but the results were not statistically significant. Additionally, this study may have missed some important factors. During a walk-through survey, we were informed that there could be considerable differences in seasonal and workday workload. MHW workers may collect more waste on Mondays, immediately after holidays, and during kimchi-making season (most Korean houses, restaurants and kimchi factories prepare kimchi for the winter in November using mainly Chinese cabbage and a large quantity of waste is generated during the trimming process). However, we did not sample during that time, hence our assessments may have underestimated worker exposure to DPM.

## Conclusions

This study is the first to assess the exposure of MHW workers to DPM using five indicators; EC, OC, TC, BC and PM 2.5. The purposes of this study were the following: to determine the exposure levels of the MHW workers to DPM, to identify an appropriate indicator of DPM exposure, and to investigate the factors influencing DPM exposure. The exposure levels of the MHW collectors were slightly higher than those of mechanics in truck repair garages and locomotive workshops, truck drivers, railroad crews and surface workers at mining facilities. Among the five DPM indicators sampled, EC was the most useful for determining MHW worker exposure. The source of EC for the MHW workers was the trash truck engines. The measurement of EC as an indicator of DPM had less interference than OC, BC and PM 2.5, and yielded a consistent and reliable exposure pattern for the various exposure factors examined.

We also investigated various occupational, vehicle and environmental factors that could significantly affect DPM exposure levels. We found that the job task, the truck engine’s European engine emission standard, and the average driving speed were the most important exposure factors for EC exposure. It should be noted that environmental regulations and auto/truck industry vehicle exhaust standards could reduce MHW worker exposure levels by increasing compliance. Therefore, the current study results should not be used to estimate past or future MHW worker exposure levels. Further study of MHW worker exposure to DPM should be conducted to include a wider range of occupational and environmental situations, and additional MHW collection procedures, daily and seasonal situations, and types of vehicle, and possibly different types of fuel.

## Supporting Information

S1 File Determination of the calibration factor for a personal aerosol monitor. (DOC)

S2 File EC, OC, TC, BC and PM 2.5 values for 72 MHW workers. (XLSX)

S1 Table Summary of study companies, work hours, waste type and number of diesel particulate matter samples. (DOC)

S2 Table Ambient background levels for each sampling period. (DOC)
