# Supplementary material for: Occupational Exposure to Diesel Particulate Matter in Municipal Household Waste Workers
Source: PLoS One. 2015 Aug 6;10(8):e0135229. doi: 10.1371/journal.pone.0135229 (PMC4527826; doi:10.1371/journal.pone.0135229)
Supplement: S1 File — (DOC) [file pone.0135229.s001.doc]

**S1 File.** **Determination of the calibration factor for a personal aerosol monitor.**

The SidePak measurements were calibrated to the PM 2.5 concentration from the corresponding gravimetric measurements. We located a SidePak monitor and three reference samplers together at the gate of truck terminal where many diesel fueled vehicles were running and idling. The samplings were performed on 20 and 23 Jan 15.

The gravimetric PM 2.5 samples were collected on a polyvinyl chloride (PVC, diameter 37 mm, pore size 5.0 μm, SKC Inc., USA) filter mounted on PM 2.5 sampler (PEM, Cat No 761-203, SKC Inc., USA) using a portable high volume pump (SKC Inc., AirChek 52, USA). The pumps drew air through sampling inlets at 2.0 Lpm. Filters were stored in desiccators before and after sampling for at least 24 hours to equilibrate temperature and humidity. Each filter was also adapted to the antistatic equipment to protect it from static electricity. Pre– and post–weighing was performed using a microbalance (Mettler Toledo Inc., XP6 Automated-S, USA ) with a sensitivity of 1μg in a weighing room where the temperature (20±5 ℃) and humidity (55±5 %) were controlled. For each date, three field blank filters were subjected to the same experimental procedures and their average ‘post-pre’ weight was subtracted from each ‘post-pre’ weight of filter.

The SidePak measurements were recalculated using the average calibration factor for each measurement as follows. Table A. presents detail gravimetric analysis data and calibration factor calculated.

| Calibration factor = | Gravimetric PM 2.5 concentration |
| --- | --- |
|  | Time integrated SidePak concentration |

Each SidePak measurement was multiplied by the calibration factor of 0.69 to estimate the true mass concentration.

Table A. Reference gravimetric PM 2.5 concentrations and calculated calibration factor

| Sampling date | Filter No. | Post-Pre weight (μg) | sampling duration (min) | Sampling volume (m^3^) | gravimetric concentration (μg/m^3^) | SidePak concentration (μg/m^3^) | Calibration factor |
| --- | --- | --- | --- | --- | --- | --- | --- |
| 20-Jan-15 | PVC-101 | 76.7 | 374 | 0.791 | 80.0 |  |  |
| 20-Jan-15 | PVC-102 | 67.0 | 374 | 0.788 | 68.0 |  |  |
| 20-Jan-15 | PVC-103 | 51.7 | 265 | 0.555 | 68.9 |  |  |
| 20-Jan-15 | Average gravimetric PM 2.5 concentration | | | | 72.3 | 91 | 0.79 |
| 23-Jan-15 | PVC-107 | 90.7 | 435 | 0.915 | 80.7 |  |  |
| 23-Jan-15 | PVC-108 | 83.3 | 435 | 0.916 | 72.6 |  |  |
| 23-Jan-15 | PVC-109 | 92.7 | 435 | 0.906 | 83.7 |  |  |
| 23-Jan-15 | Average gravimetric PM 2.5 concentration | | | | 79.0 | 134 | 0.59 |
| Average Calibration Factor | | | | | | | 0.69 |
