# Supplementary material for: Occupational Exposure to Diesel Particulate Matter in Municipal Household Waste Workers
Source: PLoS One. 2015 Aug 6;10(8):e0135229. doi: 10.1371/journal.pone.0135229 (PMC4527826; doi:10.1371/journal.pone.0135229)
Supplement: S1 Table — (DOC) [file pone.0135229.s003.doc]

**S1 Table. Summary of study companies, work hours, waste type and number of diesel particulate matter samples.**

| City | Company | Sampling date | Work hour | Type of  waste | # of Truck Surveyed | Payload capacity  (ton) | Number of workers sampled | | | | | |
| --- | --- | --- | --- | --- | --- | --- | --- | --- | --- | --- | --- | --- |
|  |  |  |  |  |  |  | EC/OC/TC | | BC | | PM 2.5 | |
|  |  |  |  |  |  |  | Clt^1)^ | Drv^2)^ | Clt | Drv | Clt | Drv |
| Goyang | A | 6/26/2014 | 04:00-13:00 | Solid | 4 | 5 | 8 | 4 | 2 | 1 | 2 | 1 |
|  | B | 7/1/2014 | 04:00-13:00 | Solid | 3 | 5 | 6 | 3 | 1 | 1 | 1 | 1 |
|  |  |  |  | Food | 2 | 5 | 2 | 1 | - | - | - | - |
|  |  | 7/2/2014 | 04:00-13:00 | Solid | 2 | 5 | 4 | 2 | 1 | 1 | 1 | 1 |
|  |  |  |  | Food | 3 | 5 | 2 | 3 | - | - | - | - |
|  |  | 7/11/2014 | 04:00-13:00 | Solid | 4 | 5 | 5 | 4 | 1 | 1 | - | 1 |
|  |  |  |  | Food | 1 | 5 | 1 | 1 | - | - | - | - |
|  | C | 7/10/2014 | 04:00-13:00 | Solid | 3 | 5 | 5 | 3 | 1 | 1 | 2 | 2 |
|  |  |  |  | Food | 2 | 5 | 2 | 2 | - | - | - | - |
| Seoul | D | 9/16/2014 | 20:00-0500 | Solid | 3 | 2.2-2.5 | 4 | 3 | 3 | 1 | 3 | 2 |
|  |  |  |  | Food | 1 | 2.2 | - | 1 | - | - | - | - |
|  | E | 9/18/2014 | 20:00-04:00 | Solid | 2 | 1.7-2.4 | 2 | 2 | 1 | 1 | 2 | 2 |
|  |  |  |  | Food | 1 | 2.5 | 1 | 1 | - | - | - | - |
| Total |  |  |  |  | 31 |  | 42 | 30 | 10 | 7 | 11 | 10 |
| ^1)^ Collector  ^2)^ Driver | | | | | | | | | | | | |
