# Supplementary material for: Occupational Exposure to Diesel Particulate Matter in Municipal Household Waste Workers
Source: PLoS One. 2015 Aug 6;10(8):e0135229. doi: 10.1371/journal.pone.0135229 (PMC4527826; doi:10.1371/journal.pone.0135229)
Supplement: S2 Table — (DOC) [file pone.0135229.s004.doc]

**S2 Table. Ambient background levels for each sampling period.**

| City | Date | Sample Time | EC  (㎍/㎥) | OC  (㎍/㎥) | TC  (㎍/㎥) | BC  (㎍/㎥) | PM 2.5  (㎍/㎥) |  |
| --- | --- | --- | --- | --- | --- | --- | --- | --- |
| Goyang | 6/26/2014 | 04:00-13:00 | 1.1 | 2.0 | 3.1 | 5.4 | 18.7 |  |
|  | 7/1/2014 | 04:00-13:00 | 1.1 | 3.7 | 4.8 | 5.8 | 19.3 |  |
|  | 7/2/2014 | 04:00-13:00 | 2.3 | 5.3 | 7.6 | 6.7 | 38.0 |  |
|  | 7/10/2014 | 04:00-13:00 | 1.3 | 3.5 | 4.8 | 5.7 | 14.0 |  |
|  | 7/11/2014 | 04:00-13:00 | 2.2 | 5.0 | 7.2 | 5.7 | 25.2 |  |
| Seoul | 9/16/2014 | 21:00-05:00 | 1.4 | 4.1 | 5.5 | 3.3 | 17.1 |  |
|  | 9/18/2014 | 21:00-04:00 | 3.0 | 4.9 | 7.9 | 4.0 | 10.0 |  |
| Abbreviations: EC: elemental carbon; OC: organic carbon; TC: total carbon; BC: black carbon; PM 2.5: particulate matter 2.5. | | | | | | | | |
